# Challenges in Diagnosing Primary Ciliary Dyskinesia in a Brazilian Tertiary Hospital

**DOI:** 10.3390/genes13071252

**Published:** 2022-07-15

**Authors:** Mariana Dalbo Contrera Toro, José Dirceu Ribeiro, Fernando Augusto Lima Marson, Érica Ortiz, Adyléia Aparecida Dalbo Contrera Toro, Carmen Silvia Bertuzzo, Marcus Herbert Jones, Eulália Sakano

**Affiliations:** 1 Department of Otolaryngology, Faculty of Medical Sciences, University of Campinas, Tessália Vieira de Camargo, 126, Cidade Universitária Zeferino Vaz, Campinas, São Paulo 13083-887, Brazil; erica.ortiz@terra.com.br (É.O.); eulalia.s@terra.com.br (E.S.); 2 Department of Pediatrics, Faculty of Medical Sciences, University of Campinas, Tessália Vieira de Camargo, 126, Cidade Universitária Zeferino Vaz, Campinas, São Paulo 13083-887, Brazil; jdirceuribeiro@gmail.com (J.D.R.); leiadalbotoro@gmail.com (A.A.D.C.T.); 3 Laboratory of Medical Genetics and Genome Medicine, Department of Medical Genetics, Faculty of Medical Sciences, University of Campinas, Tessália Vieira de Camargo, 126, Cidade Universitária Zeferino Vaz, Campinas, São Paulo 13083-887, Brazil; fernandolimamarson@hotmail.com (F.A.L.M.); bertuzzo@unicamp.br (C.S.B.); 4 Laboratory of Human and Medical Genetics, Laboratory of Cell and Molecular Tumor Biology and Bioactive Compounds, São Francisco University, Avenida São Francisco de Assis, 218, Jardim São José, Bragança Paulista, São Paulo 12916-900, Brazil; 5 Department of Pediatrics, Pontifical Catholic University of Rio Grande do Sul, Av. Ipiranga, 6681, Porto Alegre 90619-900, Brazil; mhjones@pucrs.br

**Keywords:** bronchiectasis, ciliary motility disorders, genetic testing, Kartagener syndrome, microscopy, sinusitis, transmission electron microscopy

## Abstract

Primary ciliary dyskinesia (PCD) causes cellular cilia motility alterations, leading to clinical manifestations in the upper and lower respiratory tract and situs abnormalities. The PCD diagnosis was improved after the inclusion of diagnostic tools, such as transmission electron microscopy and genetic screening; however, the PCD screening is a challenge yet. In this context, we aimed to describe the clinical, genetic, and ultra-ciliary characteristics in individuals with clinical suspicion of PCD (cPCD) from a Brazilian Tertiary Hospital. An observational study was carried out with individuals during the follow-up between 2011 and 2021. The individuals were submitted to clinical questionnaires, transmission electron microscopy, and genetic screening for pathogenic variants in PCD-related genes. Those patients were classified according to the degree of suspicion for PCD. In our study, we enrolled thirty-seven cPCD individuals; 20/37 (54.1%) had chronic rhinosinusitis, 28/37 (75.6%) had bronchiectasis, and 29/37 (78.4%) had recurrent pneumonia. A total of 17/37 (45.9%) individuals had transmission electron microscopy or genetic confirmation of PCD; 10 individuals had at least one positive pathogenic genetic variant in the PCD-related genes; however, only seven patients presented a conclusive result according to the American College of Medical Genetics and Genomics and the Association for Molecular Pathology with two pathogenic variants in homozygous or compound heterozygous. The median age at diagnosis was 13 years, and the median time between suspicion and diagnosis was four years. Sixteen patients had class I electron microscopy alterations, seven had class II alterations, and 14 had normal transmission electron microscopy according to the international consensus guideline for reporting transmission electron microscopy results in the diagnosis of PCD (BEAT-PCD TEM Criteria). Genetic screening for pathogenic variants in PCD-related genes and transmission electron microscopy can help determine the PCD diagnosis; however, they are still unavailable to all individuals with clinical suspicion in Brazil. We described ultrastructural alterations found in our population along with the identification of pathogenic variants in PCD-related genes.

## 1. Introduction

Primary ciliary dyskinesia (PCD) is a disease in which alterations in the cellular cilia motility lead to clinical manifestations, such as situs inversus, bronchiectasis, upper respiratory tract infections, and infertility [1,2]. Several pathogenic genetic variants in PCD-related genes are responsible for modifications in ciliary ultrastructure and function, resulting in a heterogenous clinical phenotype [3]. In the lower respiratory tract, the malfunction of the ciliary clearance can create an appropriate environment for acute recurrent infection, remodeling of the airways, and deteriorated lung function [4]. This motility dysfunction also increases the upper respiratory symptoms and the risk for chronic rhinosinusitis, chronic rhinitis, and chronic and acute otitis [5]. Moreover, laterality situs defects and infertility are commonly present in patients with PCD [1].

Although it has been 45 years since Afzelius described the ultrastructure ciliary alteration in individuals with bronchiectasis, sinusitis, and situs inversus, clinicians still face difficulties diagnosing PCD individuals [6,7]. The American Thoracic Society (ATS) and the European Respiratory Society (ERS) differ in their algorithm for diagnosis, and even other adaptations of this algorithm have been reported [8,9]. In addition, the lack of one gold standard test and the presence of PCD phenotype heterogeneity justifies the need to apply a different number of tools to establish a confirmatory diagnosis in those algorithms [7,8].

The challenges in diagnosing start with the selection of the individuals that should initiate investigation because clinical criteria can differ for authors [10,11,12]. For example, there is no consensus on predictive clinical questionaries such as PICADAR (PrImary CiliAry DyskinesiA Rule) questionnaire, ATS clinical screening questionnaire (ATS-CSQ), and the clinical index questionnaire [10,11,12]. Commonly used tools such as transmission electron microscopy (TEM), high-speed video microscopy, and nasal nitric oxide can be implemented in the PDC diagnosis workflows; besides that, these tools are complex, expensive, and have a considerable rate of false negatives results [1,2,13]. In addition, genetic screening for pathogenic variants in PCD-related genes of those individuals can be a solution as it is less expensive than other tools and may become even cheaper in the future. Moreover, it is a straightforward test that can be done without patients’ collaboration. However, its sensitivity is not yet known as we are not entirely aware of all genetic variants involved with the PCD disease [14,15].

In the International PCD cohort (iPCD) formed by majorly high-income countries, 30% of individuals had ambiguous or did not conclude a diagnostic algorithm [16]. The diagnostic tests are even more unavailable and challenging to perform in low-income countries and remote regions [17]. The delay in suspicion and diagnosis of this disease can corroborate with a poorer prognosis because early identification and treatment of infectious exacerbations can avoid lung structural damage in PCD individuals. Concomitantly, late diagnosis is associated with decreased lung function [18,19].

This study aimed to describe the results of the diagnostic tools (TEM, clinical questionnaires, and genetics testing) in individuals with clinical suspicion of PCD (cPCD) from a Brazilian Tertiary Hospital.

## 2. Materials and Methods

An observational study was carried out with patients during the follow-up at the Hospital de Clínicas of the University of Campinas (HC-Unicamp) due to cPCD. All the participants and their guardians signed the terms of consent. The ethics committee of the University of Campinas approved the study (CAAE: #31498020.8.0000.5404 and #48630115.0.2001.5404).

Individuals from the otorhinolaryngology clinics followed from 2011 to 2021 with cPCD were invited to participate in the research. cPCD individuals were referred by other otorhinolaryngologists, pneumologists, and pediatricians from the institution. The inclusion criteria were based on clinical characteristics of PCD described on the ERS task force: defects of laterality, positive family history of PCD, persistent rhinorrhea, chronic rhinitis, neonatal respiratory failure, productive cough, bronchiectasis, chronic otitis (chronic otitis media, serous otitis media, and conductive hearing loss), chronic rhinosinusitis, and infertility [20]. Subjects diagnosed with cystic fibrosis, α-1-antitrypsin deficiency, immunodeficiencies, and other clinical conditions that may mimic clinical alterations found on PCD were not included or excluded.

All individuals responded to a clinical form containing demographic data, characteristic symptoms of PCD, and personal history. The individuals evaluated after 2016 responded to the PICADAR questionnaire and ATS-CSQ [10,11].

All individuals were classified with low, moderate, or high suspicion for PCD according to the following criteria: (low suspicion) individuals with recurrent pneumonia or non-atopic severe asthma and upper respiratory tract infections; (moderate suspicion) individuals with bronchiectasis and sinusitis or repetition pneumonia and familiar positive history of PCD; and (high suspicion) individuals with bronchiectasis and either laterality defect or sperm defects.

For nasal evaluation, the individuals underwent nasal endoscopy. The main alterations as mucosa edema, nasal polyps, septal deviation, inferior turbinate hypertrophy, nasal secretion, and adenoid hypertrophy, were documented. Individuals who had acute upper airway infections on the collection day were rescheduled after 30 days.

Evaluation of TEM in the institution started in 2012; the ciliated epithelial tissue was collected through cytological brushing of the inferior turbinate. The material was placed in a container with a 3% glutaraldehyde fixative solution maintained at the temperature of 4 ºC for three hours. The biopsy was processed, washed, and placed in a container with a phosphate buffer. The samples were analyzed by two researchers (MDCT and EO) according to the “Better Experimental Approaches to Treat Primary Ciliary Dyskinesia” criteria (BEAT-PCD TEM criteria) from ERS [21]. 

The BEAT-PCD-TEM criteria consist of [class I alteration] hallmark defects such as more than 50% of axonemes with outer dynein arm (ODA) defects with or without inner dynein arm (IDA) defects or microtubular disorganization (MD) with IDA defects; and [class II alteration] cilia alterations that confirm PCD diagnosis in the presence of other supporting evidence which includes: central complex (CC) defects, mislocalization of basal bodies with few or no cilia (Oligocilia), MD defect with IDA present or ODA defect with or without IDA defect in 25–50% of cross-sections. 

Ultrastructure changes were based on observation in at least 100 cilia, being evaluated in cross-sections [20]. Abnormalities found in less than 10% of the cilia were considered within the normal range [22]. It was described that all alterations were found in the cilia’s ultrastructure and organization, such as the absence of the IDA and ODA, translocations and absences of central microtubules, compound cilia, ciliary disorientation, and alterations in peripheral and central microtubules [23,24].

Genetic variants were analyzed according to previously published data [19]. In brief, the venous blood was collected from individuals and transported to the Molecular Genetics Laboratory of the Faculty of Medical Sciences/Unicamp. The material was processed for DNA extraction at the laboratory using the FlexiGene DNA Kit extraction kit (Qiagen^®^, Valencia, CA, USA). After the DNA extraction was quantified in Qubit 2.0 (Life Technologies^®^, São Paulo/SP, Brazil) and being in accordingly, the sample was sent for a DNA panel gene (TruSeq^®^ 202 amplicon custom panel, San Diego, CA, USA) sequencing. The genetic sequencing was performed on the MySeq platform (Illumina^®^, San Diego, CA, USA) following three steps: (i) Creation of the DNA library; (ii) equipment sequencing to generate the DNA sequencing; and (iii) construction of the final DNA sequence by software analysis. Human Genome 19 (hg19) was used as the base genome. After the initial description of the variants, it was assigned to be associated with the disease by comparison with previously published data. The classification according to pathogenicity was done using the definition from the American College of Medical Genetics and Genomics and the Association for Molecular Pathology [25]. We considered a positive diagnosis by the genetic test the presence of two pathogenic variants in the same PCD-related gene homozygous (equal variants) or compound heterozygous (different variants in the same gene) patients. The presence of only one pathogenic variant in the PCD-related gene was associated with inconclusive genetic testing for the diagnosis. The positive genetic testing with only one pathogenic variant only was considered when we had the pathogenic variant in an X-linked gene for male patients only.

The follow PCD-related genes were analyzed in study: Armadillo Repeat Containing (*ARMC4*); Chromosome 21 Open Reading Frame 59 (*C21ORF59*); Coiled-Coil Domain Containing 103 (*CCDC103*); Coiled-Coil Domain Containing 114 (*CCDC114*); Coiled-Coil Domain Containing 151 (*CCDC151*); Coiled-Coil Domain Containing *39 (CCDC39)*; Coiled-Coil Domain Containing 40 (*CCDC40*); Coiled-Coil Domain Containing 65 (*CCDC65*); Cyclin O (*CCNO*); Dynein, Axonemal, Assembly Factor 1 (*DNAAF1*); Dynein, Axonemal, Assembly Factor 2 (*DNAAF2*); Dynein, Axonemal, Assembly Factor 3 (*DNAAF3*); Dynein, Axonemal, heavy Chain 11 (*DNAH11)*; Dynein, Axonemal, heavy Chain 5 (*DNAH5)*; Dynein, Axonemal, Intermediate Chain 1 (*DNAI1*); Dynein, Axonemal, Intermediate Chain 2 (*DNAI2*); Dynein, Axonemal, Light Chain 1 (*DNAL1*); Dynein Regulatory Complex Subunit 1 (*DRC1*); Dyslexia Susceptibility 1 Candidate 1 (*DYX1C1*); HEAT Repeat Containing 2 (*HEATR2*); Axonemal Central Pair Apparatus Protein (*HYDIN*); Leucine Rich Repeat Containing 6 (*LRRC6*); NME/NM23 Family Member 8 (*NME8*); Oral-Facial-Digital Syndrome 1 (*OFD1*); Retinitis Pigmentosa Gtpase Regulator (*RPGR*); Radial Spoke Head 1 Homolog (*Chlamydomonas*) (*RSPH1*); Radial Spoke Head 4 Homolog A (*Chlamydomonas*) (*RSPH4A*); *RSPH9*; Sperm Associated Antigen 1 (*SPAG1*); Zinc Finger, MYND-Type Containing 10 (*ZMYND10*); and Coiled-Coil Domain Containing 164 (*CCDC164*). In addition, genetic sequencing of the Cystic Fibrosis Transmembrane Conductance Regulator (*CFTR*) gene was performed to exclude the cystic fibrosis diagnosis. 

All individuals had their clinical and diagnosis data described in an excel sheet and classified as PCD diagnosis, excluded, or dubious diagnosis. A retrospective review of the charts allowed the researchers to double-check for any clinical and diagnosis alterations through the years. Moreover, the age at suspicion and the age at diagnosis were recorded. It was evaluated which tools were used to diagnose these patients [clinical characteristics, TEM, genetics testing, PICADAR, and ATS-CSQ]. It was considered a PICADAR > 6 points and an ATS-CSQ > 2 points as cutoffs for suspicion of PCD as previously reported in the literature [10,11,26].

The descriptive analysis was performed using categorical data by absolute and relative frequency. Numeric data are presented by the median, minimum, and maximum values, and interquartile range. The normality of the numerical data was evaluated by the following techniques: (I) measurement analysis descriptive for central tendency; and (II) method by statistical test (normality tests): Kolmogorov–Smirnov and Shapiro–Wilk. The statistical analysis was done using the Kruskal Wallis test to compare the age among patients grouped by cPCD. The statistical analysis was performed using the Statistical Package for the Social Sciences software (IBM SPSS Statistics for Macintosh, Version 27.0, São Paulo, SP, Brazil). The level of significance considered was 0.05.

## 3. Results

**Figure 1:**
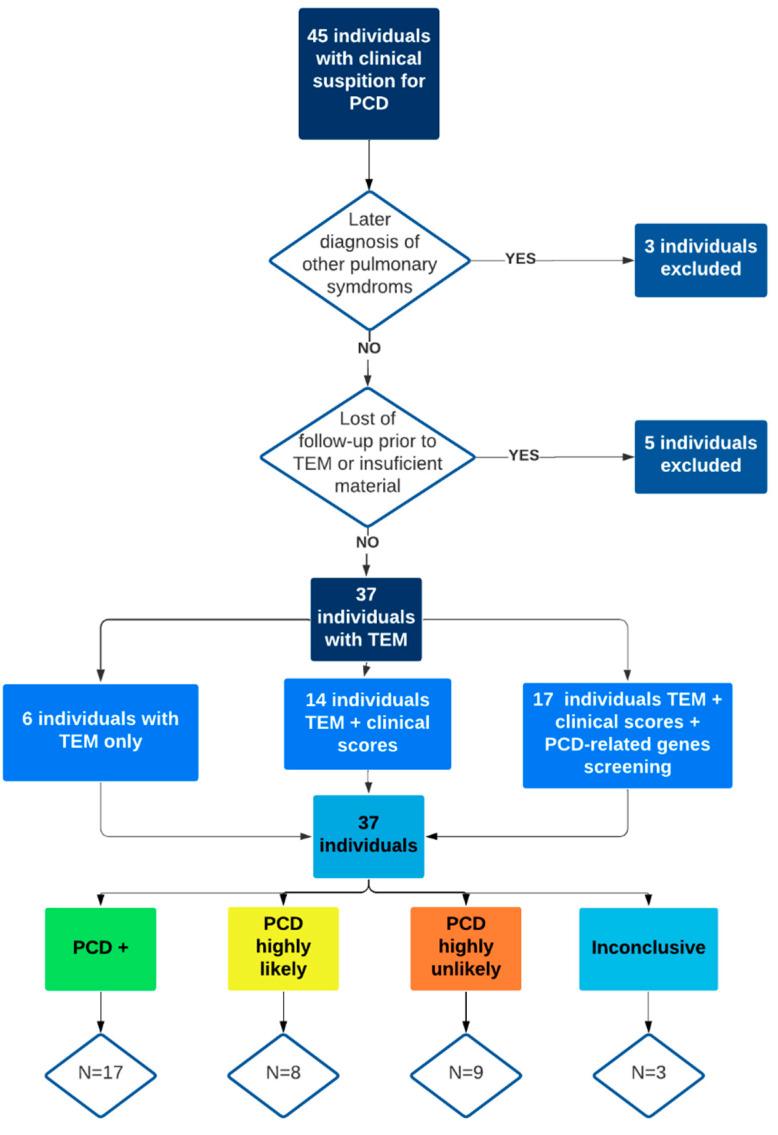
Included patients’ algorithm and division by the degree of clinical suspicion. PCD: primary ciliary dyskinesia, TEM: transmission electron microscopy, N: number of individuals.

A total of 45 cPDC individuals were initially included. Three individuals were excluded due to later diagnosis of cystic fibrosis and Willian Campbell syndrome. Two patients were excluded after the loss of follow-up before any diagnostic tests were concluded. Three individuals had insufficient material for TEM analysis. Of the remaining 37 individuals, six did not answer the PICADAR questionnaire and ATS-CSQ because of the implementation of this tool after 2016, and 14 did not undergo genetic testing due to the low financial support. Seventeen individuals had the complete protocol done (Figure 1).

In our study cohort, 25 (67.6%) individuals were male; 20 (54.1%) had chronic rhinosinusitis, 28 (75.7%) had bronchiectasis, and 29 (78.4%) had recurrent pneumonia. Table 1 summarizes the clinical alterations and the individuals’ classification in cPCD. The age at suspicion and diagnosis of the individuals according to the degree of cPCD suspicion are summarized in Table 2. The median age at diagnosis was 13 years, and the median time between suspicion and diagnosis was four years, and the mean time 7.5 years. The PICADAR score predicted 55% of the positive confirmed diagnosis, and ATS-CSQ was positive in 77.8% of the cases.

Table 3 summarizes the diagnostic tests, clinical scores, and age at suspicion and diagnosis of PCD. A total of 17 (45.9%) individuals had either TEM or genetic confirmation of PCD. When considering the degree of clinical suspicion, out of the 13 individuals in the high suspicion group, 10 (76.9%) had a PCD diagnosis, and three (23.1%) had a highly likely PCD diagnosis. In the moderate suspicion group, which enrolled 16 individuals, five (31.6%) had a PCD positive diagnosis, five (31.6%) had a highly likely PCD diagnosis, four (25%) had a highly unlikely PCD diagnosis, and two (12.5%) had inconclusive tests result; this group was the most heterogeneous one. In the individuals in the low suspicion group, two (25%) individuals had a PCD positive diagnosis, five (62.5%) had a highly unlikely PCD diagnosis, and one (12.5%) had an inconclusive PCD diagnosis.

**Figure 2:**
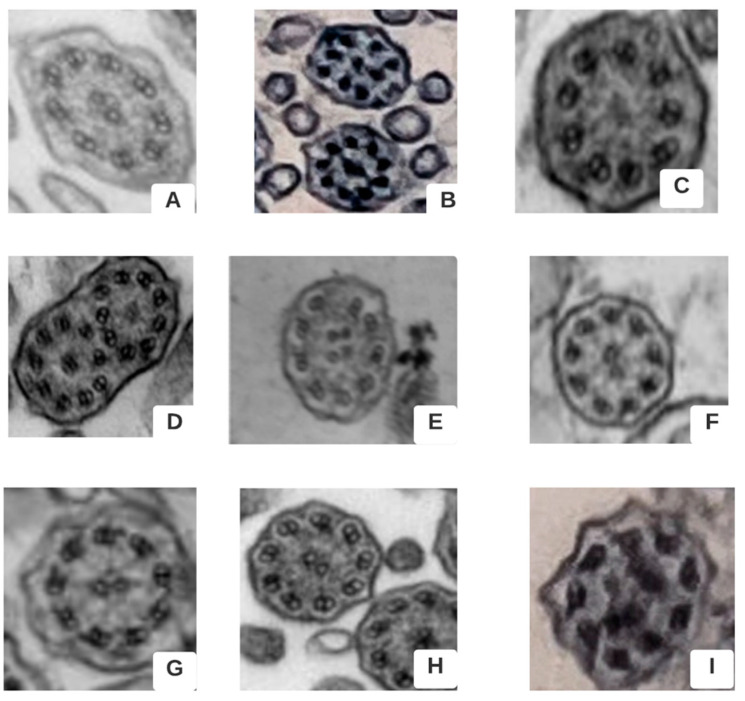
Transmission electron microscopy (TEM) findings as described in the “Better Experimental Approaches to Treat Primary Ciliary Dyskinesia” criteria (BEAT-PCD TEM criteria) from ERS [21] and TEM images of nasal brushing of patients from the study. (**A**) Normal ultrastructure (Case. 8). (**B**) TEM showing absence of inner and outer dynein arm combined with microtubular disorganization (Case 13). (**C**,**D**) Absent inner dynein and outer dynein arm and compound cilia (Case 15). (**E**) Two pairs of central microtubules (Case 29). (**F**) Absent inner dynein arm (Case 19). (**G**) Absent inner dynein arm (Case 21). (**H**) Absent inner dynein and outer dynein arm (Case 27). (**I**) Absence of inner dynein arm combined with microtubular disorganization and central complex defect (Case 28).

A total of 17 individuals underwent genetic screening for pathogenic variants in the PCD-related genes. Of these, 10 had at least one positive pathogenic variant for PCD (half patients in homozygosis–two pathogenic variants resulting in a conclusive result, two patients with a compound heterozygosis–two pathogenic variants resulting in a conclusive result, and three patients with only one pathogenic variant–one pathogenic variant resulting in an inconclusive result), two had the *CFTR* in heterozygosis, and five had negative results for the genes included in the DNA panel gene. Table 4 shows the individuals with positive genetic variants for PCD, their pathogenic variants, proteins involved, and the expected ciliary ultrastructure alteration. Figure 2 shows TEM findings according to the BEAT-PCD-TEM criteria [21]. 

## 4. Discussion

Diagnosing PCD is a challenge often explained by the low sensitivity and specificity of the diagnostic tests used, concomitantly with the presence of phenotype and genotype variability [8]. However, in our data, implementing genetic testing in our practice improved the number of individuals with a definitive diagnosis. The establishment of genetic testing was beneficial in confirming PCD in our study cohort. Moreover, it could help with genetic counseling in the future. This tool offers an accurate diagnosis with no age restrictions and is potentially more cost-efficient [15]. On the other hand, at least 45 PCD genes have so far been described, but there is still a significant number of individuals with positive PCD diagnosis and inconclusive genetic testing. Curiously, PCD can be caused by pathogenic variants in genes encoding proteins that are necessary for ciliary function but with normal ultrastructure [14].

However, with limited resources, countries have additional difficulty because of the need for expensive equipment and highly trained professionals [17], and many regions of the world still suffer from a probably underestimated number of cases [27]. It was established that in European countries, the number of diagnosed cases and the age at diagnosis correlated with the government’s health expenditure [28]. In Brazil, the national health care system still does not cover the use of any diagnostic tool for PCD, and the only individuals with a definitive diagnosis are those enrolled in research projects. 

Individuals with situs inversus are generally diagnosed earlier due to a higher suspicion of ciliopathies [28,29]. An international study showed that 37% of PCD individuals were referred to specialists for diagnosis after at least 40 visits [30]. In our study, there were no differences in the median age of individuals with clinical low, moderate, or high suspicion. Furthermore, we had a higher median age of diagnosis than reported in the literature [28,30], which is probably explained by the damming of cases in the last couple of decades. A late diagnosis of this condition can impact a more significant number of lower respiratory exacerbations and *Pseudomonas aeruginosa* colonization, which may be associated with pulmonary structure and function deterioration [18]. Moreover, a previous study demonstrated that individuals with an earlier diagnosis of PCD presented better health-related quality of life, highlighting the impact of prompt health interventions [31].

Deciding which individuals should undergo a detailed investigation is still a question to be answered. In our study, we had two patients with low clinical suspicion for PCD with a confirmed diagnosis, which confirms the disease is heterogenous and varies over the years. Therefore, relying solely on clinical features and classic disease symptoms can be misleading [4]. Screening questionaries such as PICADAR and the ATS-CSQ, along with the clinical index, were created to facilitate referral from primary doctors [10,11,12]. Although all have proven to have good sensibility compared to other diagnostic tools, they still need validation, as they were created for a specific population [10,11,12]. In our study, almost all individuals with high suspicion of cPCD had their diagnosis confirmed. However, in the low and moderate groups, the diagnosis was very heterogenous; in these groups, PICADAR failed to predict diagnosis in most PCD+ individuals. 

The PICADAR was validated in a pediatric population, and cough was the most important predictive factor; this tool in a mixed-aged population referred to the otorhinolaryngology outpatient clinic may not be so valuable. The ATS–CSQ was a more straightforward and reliable tool in our study but still not flawless. For this reason, individuals with low and moderate suspicion should look closely as they often demand more effort to ensure diagnosis. Physicians should be aware of patients with severe or atypical symptoms and individually assess each patient’s medical history [20]. Furthermore, in adult individuals, physicians are often more worried about deteriorating lung function and determining a specific diagnosis [30]. Considering the lack of clinical diagnostic tools for this population, along with the lower level of suspicion, there is a great chance that adult individuals are underdiagnosed.

Genetic testing can improve the number of individuals with PCD diagnosis. Concerning the genetic analysis, the *DNAH5* pathogenic variants are the most common gene alterations in PDC individuals, and these variations result in ODA defects, the ODA being the main mechanical force responsible for cilia movement, and the absence of this protein will likely result in an immotile cilium [3,32]. Two distinct ODA complexes can be found within the ciliated epithelium; although they have different distributions along the cilia axoneme, both contain DNAH5 [33]. The two individuals in our study with *DNAH5* variants were heterozygous (one had only one variant, and the other was a compound heterozygote), and none had isolated ODA defects. As one of our patients with ODA+IDA defects, Faily et al. reported that 27% of its cohort with ODA + IDA defects presented *DNAH5* variants [34]. Interestingly, one of our individuals did not have an ODA defect which can be explained by *DNAH5* not being the gene causing PCD, or TEM may have missed ODA defects that may be only present in the distal part of the axoneme (defect of only one ODA Type 2) [33].

According to the literature, *DNAH11* pathogenic gene variants, despite encoding a protein of the ODA, are associated with normal ciliary ultrastructure [35]. Cases of PCD with *DNAH11* variants can be identified through abnormal hyperkinetic ciliary beat patterns in high-speed video microscopy immunofluorescence and TEM [36]. As with the *DNAH11*, *DNA12* also encodes a protein in the ODA; the individuals with this alteration may present with modified immunofluorescence microscopy and high-speed video microscopy with an irregular ciliary beat. Moreover, the *CCDC151* encodes an anchoring protein of the external dynein arm, and individuals with this genetic variant have laterality defects and severe cardiac malformations [37]. Curiously, both individuals in our study with pathogenic variants in the *CCDC151* had situs inversus; however, only one presented an ODA defect, along with other ultrastructural alterations, and the other had normal ciliary ultrastructure. *ARMC4* pathogenic variants also caused abnormal ODA docking, but in this case, it is more prominent in the distal ciliary axoneme, and it is associated with left-right laterality defects; similarly, in our study, the individual that presented the pathogenic variant had dextrocardia along with bronchiectasis and chronic rhinosinusitis [3,38].

The *CCDC40* is related to the absence of the IDA with or without axoneme disorganization [39]. In our study, both individuals with *CCDC40* pathogenic variants had IDA defects along with axoneme disorganization, chronic rhinosinusitis, bronchiectasis, and recurrent pneumonia, and one of them also presented dextrocardia. In the literature, *CCDC40* was linked with a worsening lung function [3]. Importantly, most genetic variants were compatible with the TEM findings in our study [2]. 

Our study demonstrated a high prevalence of IDA defects that did not correlate with the genetic variants. The IDA defect can be present transiently in TEM analyses, especially associated with inflammation, and sometimes be present after cell culture [40]. Moreover, IDA is less dense than other intraciliary structures and may be challenging to visualize [40]. Therefore, although commonly present in our studies, the absence of IDA was not considered diagnostic for PCD, as recommended in the international consensus [21,40].

The guidelines established by the ERS and ATS use additional tools such as nNO and high-speed optical microscopy [1,2]. In our center, we currently do not have the availability of these tools that could help in doubtful cases, contributing to a faster diagnosis of these patients. However, this lack of resources is evident in our country and other developing and undeveloped countries. Getting attention to this condition is essential for us to think about simple and cost-effective guidelines for the future that cover populations around the world. In this sense, genetic evaluation can be essential [15,17].

Our study demonstrated a diagnostic profile for a rare disease and its practical evolution over the years. It also reveals a genetic and TEM profile in a south American population that is still poorly studied. The main limitations included loss of follow-up of individuals during the implementation of new tools and the limitations on diagnostic tools provided by our healthcare system. Genetic testing, for instance, was performed in search of only 31 of the 45 known PCD-related genes, and only 17 out of the 37 patients were sequenced due to financial resources. Moreover, we could not confirm phase by first-degree testing in the two patients with variants in two different genes. However, these diagnostic difficulties related to costs, late evaluation of patients, and lack of equipment and qualified professionals to evaluate these complex tests reflect the reality of most countries outside the United States-Europe axis. This study brings light to cPCD and urges an earlier diagnosis of the disease through awareness of the primary physicians that first attend to those individuals.

## 5. Conclusions

This study highlights the difficulties of diagnosing PCD in a developing country. Genetic variants analyses and TEM can help determine this diagnosis; however, they are still unavailable to all patients. We described ultrastructural alterations found in our population along with genetic analyses variants.

## Figures and Tables

**Table 1:** Clinical characteristics and time to diagnosis in individuals with low, moderate, and high suspicion for primary ciliary dyskinesia (PCD).

	Case	PCD Diagnosis	Time to Diagnosis *	CRS	NP	CR	Asthma	Bronchiectasis	LD	RP	CO	FD	Consanguinity
*Low* *clinical suspicion*	**1**	PCD+	4	-	-	+	-	+	-	-	+	-	-
	**2**	PCD highly unlikely		-	-	+	+	-	-	+	+	-	-
	**3**	Inconclusive		-	-	+	-	-	-	+	-	-	-
	**4**	PCD+	0	-	-	+	+	-	-	+	-	-	-
	**5**	PCD highly unlikely		-	-	-	-	+	-	+	-	-	-
	**6**	PCD highly unlikely		+	+	+	+	-	-	-	-	-	-
	**7**	PCD highly unlikely		-	-	+	+	+	-	+	-	-	-
	**8**	PCD highly unlikely		-	-	+	+	-	-	+	-	-	-
*Moderate clinical suspicion*	**9**	PCD highly unlikely		-	-	+	+	+	-	+	+	-	-
	**10**	PCD highly likely		+	+	-	-	+	-	+	+	-	+
	**11**	PCD highly likely		+	-	+	+	+	-	+	+	-	-
	**12**	PCD highly unlikely		-	-	+	+	+	-	+	+	-	-
	**13**	PCD+	3	+	-	-	-	+	-	+	+	-	-
	**14**	PCD+	1	+	-	+	+	+	-	+	+	-	-
	**15**	PCD+	1	+	-	-	-	+	-	+	+	-	-
	**16**	PCD highly unlikely		+	+	-	+	+	-	-	-	-	-
	**17**	PCD highly likely		-	-	+	+	+	-	-	-	-	-
	**18**	PCD+	2	+	+	-	-	+	-	+	-	-	-
	**19**	PCD highly likely		-	-	-	+	-	-	+	-	-	-
	**20**	PCD highly unlikely		-	-	+	+	+	-	+	+	-	-
	**21**	PCD highly likely		+	+	-	-	+	-	+	-	-	-
	**22**	PCD+	5	-	-	+	-	+	-	+	-	-	-
	**23**	Inconclusive		-	-	-	-	-	-	+	-	-	-
	**24**	Inconclusive		+	-	+	-	+	-	+	+	-	-
*High clinical suspicion*	**25**	PCD+	14	+	-	-	-	+	-	-	-	+	-
	**26**	PCD highly likely		+	+	-	-	+	+	+	-	+	-
	**27**	PCD+	7	+	+	-	-	+	+	-	+	-	-
	**28**	PCD+	12	+	-	-	-	+	+	+	-	-	-
	**29**	PCD+	19	+	-	-	-	+	+	-	-	-	-
	**30**	PCD+	24	-	-	+	+	+	+	+	+	-	+
	**31**	PCD+	1	+	-	-	+	+	-	-	-	+	-
	**32**	PCD highly likely		+	-	-	-	+	+	+	+	-	-
	**33**	PCD highly likely		+	-	-	-	-	+	+	+	-	-
	**34**	PCD+	11	-	-	+	-	-	+	+	+	-	-
	**35**	PCD+	0	-	-	-	-	+	+	+	+	-	+
	**36**	PCD+	2	+	+	-	-	+	+	+	+	-	-
	**37**	PCD+	22	+	-	-	-	+	+	+	-	+	-

CRS: Chronic rhinosinusitis, NP: nasal polyps, CR: chronic rhinitis, CO: chronic otitis, RP: recurrent pneumonia, LD: laterality defect, FD: fertility disorder, +: positive result; -: negative result. * Time between suspicion and diagnosis in individuals with confirmation of PCD by TEM or genetic screening for PCD-related genes. All individuals were classified with low, moderate, or high suspicion for PCD according to the following criteria: (low suspicion) individuals with recurrent pneumonia or non-atopic severe asthma and upper respiratory tract infections; (moderate suspicion) individuals with bronchiectasis and sinusitis or repetition pneumonia and familiar positive history of PCD; and (high suspicion) individuals with bronchiectasis and either laterality defect or sperm defects.

**Table 2:** The median age of individuals at suspicion and confirmed diagnosis, separated by degree of clinical suspicion for primary ciliary dyskinesia.

	**N**	Median Age Suspicion *	**Minimum**	**Maximum**	**P25**	**P75**
***Low suspicion***	8	13	13	13	13	13
***Moderate suspicion***	16	9	2	48	8	12
***High suspicion***	13	3.5	0	46	1	15
**Total**	37	8	0	48	1	13
	**N**	Median age diagnosis **	**Minimum**	**Maximum**	**P25**	**P75**
***Low suspicion***	2	15	13	17	13	17
***Moderate suspicion***	5	11	7	50	10	13
***High suspicion***	10	19.5	2	60	9	32
**Total**	17	13	2	60	10	25

* *p* = 0.309; ** *p* = 0.760. N: number of individuals with clinical suspicion for primary ciliary dyskinesia, P25: percentile 25%, P75: percentile 75%. The statistical analysis was done using the Kruskal Wallis test. An α error of 0.05 was used in all statistical analyses.

**Table 3:** Individuals with clinical suspicion of primary ciliary dyskinesia (PCD), degree of clinical suspicion, and results of diagnostic tests (TEM, clinical scores, and genetic testing).

	Case	Sex	Age at Suspicion	TEM ^a^	Genetics	PICADAR ≥ 7	ATS-CSQ ≥ 2	PCD Diagnosis	Age at Diagnosis
*Low clinical* *suspicion*	**1**	F	13	Class I	-	-	-	PCD+	17
	**2**	M	8	Normal	N/D	-	+	PCD highly unlikely	
	**3**	M	9	Class II	N/D	-	-	Inconclusive	
	**4**	F	13	Class I	N/D	-	+	PCD+	13
	**5**	M	7	Normal	N/D	N/D	N/D	PCD highly unlikely	
	**6**	M	15	Normal	N/D	N/D	N/D	PCD highly unlikely	
	**7**	M	10	Normal	N/D	-	-	PCD highly unlikely	
	**8**	M	15	Normal	N/D	-	-	PCD highly unlikely	
*Moderate clinical* *suspicion*	**9**	F	16	Normal	N/D	-	+	PCD highly unlikely	
	**10**	M	0	Class II	N/D	+	+	PCD highly likely	
	**11**	F	9	Class II	N/D	-	+	PCD highly likely	
	**12**	M	16	Normal	-	-	+	PCD highly unlikely	
	**13**	F	8	Class I	+	+	+	PCD+	11
	**14**	M	12	Class I	N/D	-	-	PCD+	13
	**15**	M	9	Class I	+	+	+	PCD+	10
	**16**	M	44	Normal	N/D	N/D	N/D	PCD highly unlikely	
	**17**	F	4	Class II	N/D	+	+	PCD highly likely	
	**18**	M	48	Class I	-	-	+	PCD+	50
	**19**	M	1	Normal	+ *	-	-	PCD highly likely	
	**20**	F	15	Normal	N/D	-	+	PCD highly unlikely	
	**21**	M	21	Normal	+ *	-	+	PCD highly likely	
	**22**	M	2	Class I	-	-	-	PCD+	7
	**23**	F	2	Normal	N/D	+	+	Inconclusive	
	**24**	F	3	Normal	N/D	-	-	Inconclusive	
*High clinical* *suspicion*	**25**	M	46	Class I	N/D	N/D	N/D	PCD+	60
	**26**	M	27	Class II	N/D	N/D	N/D	PCD highly likely	
	**27**	M	25	Class I	+	+	+	PCD+	32
	**28**	M	0	Class I	+	+	+	PCD+	12
	**29**	F	15	Class I	-	+	+	PCD+	34
	**30**	M	1	Normal	+	+	+	PCD+	25
	**31**	M	28	Class I	N/D	N/D	N/D	PCD+	28
	**32**	M	0	Class II	-	+	+	PCD highly likely	
	**33**	F	8	Class II	+ *	+	+	PCD highly likely	
	**34**	M	6	Class I	N/D	-	+	PCD+	17
	**35**	F	1	Class I	-	+	+	PCD+	2
	**36**	M	7	Class I	+	+	+	PCD+	9
	**37**	M	0	Class I	+	-	+	PCD+	22

F: female, M: male, PICADAR: PrImary CiliAry DyskinesiA Rule, TEM: transmission electron microscopy, N/D: not done, ATS-CSQ: American Thoracic Society clinical screening questionnaire, +: positive result; -: negative result. ^a^, The BEAT-PCD-TEM criteria consist of (class I alteration) hallmark defects such as more than 50% of axonemes with outer dynein arm (ODA) defects with or without inner dynein arm (IDA) defects or microtubular disorganization (MD) with IDA defects; (class II alterations) cilia alterations that confirm PCD diagnosis in the presence of other supporting evidence which includes central complex defects, mislocalization of basal bodies with few or no cilia (Oligocilia), MD defect with IDA present or ODA defect with or without IDA defect in 25–50% of cross-sections. * Positive for one pathogenic genetic variant only. All individuals were classified with low, moderate, or high suspicion for PCD according to the following criteria: (low suspicion) individuals with recurrent pneumonia or non-atopic severe asthma and upper respiratory tract infections; (moderate suspicion) individuals with bronchiectasis and sinusitis or repetition pneumonia and familiar positive history of PCD; and (high suspicion) individuals with bronchiectasis and either laterality defect or sperm defects.

**Table 4:** Individuals with genetic variants positive for primary ciliary dyskinesia, their clinical and transmission electron microscopy findings.

Case	Clinical Findings	TEM Findings	Gene	Protein	c.DNA	code	*Alleles*	Expected Ultrastructural Alterations *
*Patients who received a definitive primary ciliary dyskinesia diagnosis by a conclusive genetic test.* *^a^*								
** 13 **	BCT, CRS, CO, RP	IDA + ODA/MD	*DNAH11*	p.Cys1597Phe	c.4790G>T	rs72657327	Hom	Normal ultrastructure/ODA defects
			*CCDC40*	p.Ala83ValfsTer84 and p.Leu872Ter	c.248delC and c.2614delC	Without id and rs775128843	Het and het	96 nm axonemal ruler: IDA+MD
** 15 **	BCT, CO, CRS, RP	IDA + ODA	*DNAH5*	p.Arg4577Ter	c.13729C>T and c.11571-1G>A	Both variants did not have an id	Het and het	ODA defects
** 27 **	BCT, CO, CRS, SI	IDA + ODA	*CCDC151*	p.His199ArgfsTer60	c.583_595dupGCGCAAAACAGAC	rs750658321	Hom	ODA docker
** 28 **	BCT, CRS, DC, RP	IDA + CCD + MD	*CCDC40*	p.Leu872Ter and p.Ala83ValfsTer84	c.2614delC and c.248delC	rs775128843 and rs397515393	Het and het	96 nm axonemal ruler: IDA+MD
** 30 **	AR, Asthma, BCT, CH, CO, RP, SI	-	*CCDC151*	p.His199ArgfsTer60	c.583_595dupGCGCAAAACAGAC	rs750658321	Hom	ODA docker
** 36 **	BCT, CO, CRS, DC, RP	IDA + ODA + CCD	*ARMC4*	p.Gln320SerfsTer44	c.958delC	Without id	Hom	ODA docker
** 37 **	BCT, CRS, RP, SD, SI	IDA + ODA	*DNAI2*	p.Arg263Ter	c.787C>T	rs137852998	Hom	ODA defects
*Patients who received an inclusive primary ciliary dyskinesia in the genetic test due the presence of only one pathogenic variant.* *^b^*								
** 19 **	Asthma, FH+, RP	IDA	*DNAH11*	p.Met1096Ile	c.3288G>A	rs575775297	Het	Normal ultrastructure/ODA defects
** 21 **	CRS, BCT, FH+, RP	IDA	*DNAH11*	p.Met1096Ile	c.3288G>A	rs575775297	Het	Normal ultrastructure/ODA defects
** 33 **	CO, CRS, RP, SI	IDA + CCD	*DNAH5*	p.Arg3885Ter	c.11653C>T	rs756032160	Het	ODA defects

AR: allergic rhinitis, FH+: positive family history, SD: sperm defects, CRS: chronic rhinosinusitis, BCT: bronchiectasis, CO: chronic otitis, SI: Situs Inversus, DC: dextrocardia, RP: recurrent pneumonia, TEM: transmission electron microscopy, IDA: inner dynein arm defect, ODA: outer dynein arm defect, CCD: central complex defect, MD: microtubular disorganization, Hom: Homozygous, Het: Heterozygous, Armadillo Repeat Containing (*ARMC4*), Coiled-Coil Domain Containing 151 (*CCDC151*); Coiled-Coil Domain Containing 40 (*CCDC40*); Dynein Axonemal Heavy Chain 5 (*DNAH5*); Dynein Axonemal Heavy Chain 11 (*DNAH11*); Dynein, Axonemal, Intermediate Chain 2 (*DNAI2*). The classification according to pathogenicity was done using the definition from the American College of Medical Genetics and Genomics and the Association for Molecular Pathology [25] ^a^, the positive diagnosis by the genetic test was done by the presence of two pathogenic variants in the same PCD-related gene. We considered the diagnosis for homozygous or compound heterozygous patients. ^b^, the presence of only one pathogenic variant in the PCD-related gene was associated with inclusive genetic testing for the diagnosis. The positive genetic testing with only one pathogenic variant occurred when we had the pathogenic variant in an X-linked gene for male patients only. Human Genome 19 (hg19) was used as the base genome. * Expected ultrastructural alterations related to genetic variants according to Lucas et al. [3].

## Data Availability

The datasets analyzed during the current study are available from the corresponding author upon reasonable request.
